# Novel mutations in *PIEZO1* cause an autosomal recessive generalized lymphatic dysplasia with non-immune hydrops fetalis

**DOI:** 10.1038/ncomms9085

**Published:** 2015-09-03

**Authors:** Elisavet Fotiou, Silvia Martin-Almedina, Michael A. Simpson, Shin Lin, Kristiana Gordon, Glen Brice, Giles Atton, Iona Jeffery, David C. Rees, Cyril Mignot, Julie Vogt, Tessa Homfray, Michael P. Snyder, Stanley G. Rockson, Steve Jeffery, Peter S. Mortimer, Sahar Mansour, Pia Ostergaard

**Affiliations:** 1 Cardiovascular and Cell Sciences Institute, St. George’s University of London, Cranmer Terrace, London SW17 0RE, UK; 2 Department of Medical and Molecular Genetics, Division of Genetics and Molecular Medicine, Kings College London School of Medicine, Guy's Hospital, London SE1 9RY, UK; 3 Division of Cardiovascular Medicine, Stanford University, Stanford, California 94305, USA; 4 Department of Genetics, Stanford University, Stanford, California 94305, USA; 5 Department of Dermatology, St. George’s Healthcare NHS Trust, London SW17 0QT, UK; 6 South West Thames Regional Genetics Unit, St. George's University of London, London SW17 0RE, UK; 7 Pathology Department, St. George's University of London, London SW17 0RE, UK; 8 Department of Haematological Medicine, King’s College London School of Medicine, King’s College Hospital, London SE5 9RS, UK; 9 Département de Génétique, APHP, GH Pitié-Salpêtrière, Centre de Référence des Déficiences Intellectuelles de Causes Rares, 75013 Paris, France; 10 West Midlands Regional Genetics Service, Clinical Genetics Unit, Birmingham Women's Hospital, Birmingham B15 2TG, UK

## Abstract

Generalized lymphatic dysplasia (GLD) is a rare form of primary lymphoedema characterized by a uniform, widespread lymphoedema affecting all segments of the body, with systemic involvement such as intestinal and/or pulmonary lymphangiectasia, pleural effusions, chylothoraces and/or pericardial effusions. This may present prenatally as non-immune hydrops. Here we report homozygous and compound heterozygous mutations in *PIEZO1*, resulting in an autosomal recessive form of GLD with a high incidence of non-immune hydrops fetalis and childhood onset of facial and four limb lymphoedema. Mutations in *PIEZO1*, which encodes a mechanically activated ion channel, have been reported with autosomal dominant dehydrated hereditary stomatocytosis and non-immune hydrops of unknown aetiology. Besides its role in red blood cells, our findings indicate that PIEZO1 is also involved in the development of lymphatic structures.

## Abstract (web summary)

Primary lymphoedema can lead to the swelling of the extremities and facial dysmorphism. Here the authors present evidence that compound heterozygous and homozygous mutations in *PIEZO1* result in an autosomal recessive form of generalised lymphatic dysplasia.

## Graphical abstract

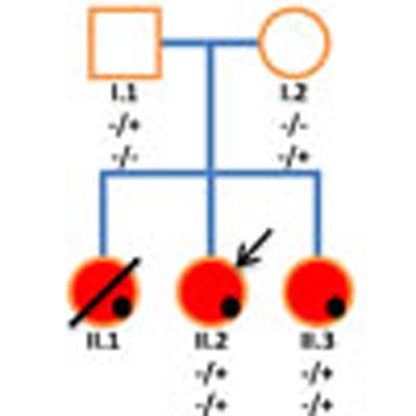

Generalized lymphatic dysplasia (GLD) is a rare form of primary lymphoedema characterized by a uniform, widespread lymphoedema affecting all segments of the body with systemic involvement such as intestinal and/or pulmonary lymphangiectasia, pleural effusions, chylothoraces and/or pericardial effusions^1^. The lymphatic dysfunction often presents prenatally as non-immune hydrops fetalis (NIHF) and the incidence of hydrops is higher than seen in other primary lymphoedemas. Other clinical features may include facial dysmorphism, possibly due to *in utero* oedema.

An autosomal recessive type of GLD has previously been reported; Hennekam Lymphangiectasia-Lymphoedema syndrome (HS) (OMIM #235510 and OMIM #616006), caused by mutations in *CCBE1* (refs 2, 3) and *FAT4* (ref. 4) explaining <50% of HS cases. Patients with HS typically have full body oedema including severe facial swelling giving these patients the very characteristic HS facial features including periorbital oedema, retrognathia, flat facial profile, high palate, gingival hypertrophy and microstomia^5^. The majority of HS patients also variably have intestinal lymphangiectasia, seizures, microcephaly, mild growth retardation and intellectual disability.

We have identified a further form of recessive GLD characterized by a high incidence of NIHF with either demise or complete resolution of the neonatal oedema but childhood onset of lymphoedema with or without systemic involvement. There is often the presence of mild facial oedema but facial cellulitis is a common and serious complication of this disorder. The facial features are not suggestive of HS and this cohort has normal intelligence and no seizures.

We performed a sequencing study and identified unreported variants in a mechanically activated ion channel, PIEZO1, that co-segregate with the disease status in the six families under study. The mutations were shown by western blot to affect expression of PIEZO1 in affected individuals. Heterozygous mutations in *PIEZO1* are reported to cause a rare form of haemolytic anaemia called dehydrated hereditary stomatocytosis (DHS), and careful examination of the blood films from three of the affected patients and five carrier parents have demonstrated subtle changes consistent with abnormalities of the red cell membrane. This suggests a phenotypic overlap between these two entities, but more analysis would be required to fully elucidate the extent of this. However, it appears that PIEZO1 is not only important for red blood cell (RBC) stability, but also in lymphatic development.

## Results

### Patient selection

Two sibling pairs and one sporadic case of GLD with no identifiable mutations in *CCBE1* were initially identified for analysis. NIHF was documented in two of these families (GLD2 and GLD3), with *in utero* demise of one sibling (GLD2:II.1, Table 1). Postnatally, the oedema associated with the hydrops resolved completely but the patients re-presented with lymphoedema of the peripheries in early childhood (Table 1). Two of the affected individuals suffer from intermittent, severe facial swelling due to recurrent cellulitis. This is rarely seen in other forms of primary lymphoedema. There was no history of haemolytic anaemia, and the immune profiles for both siblings were entirely normal.

**Figure 1:**
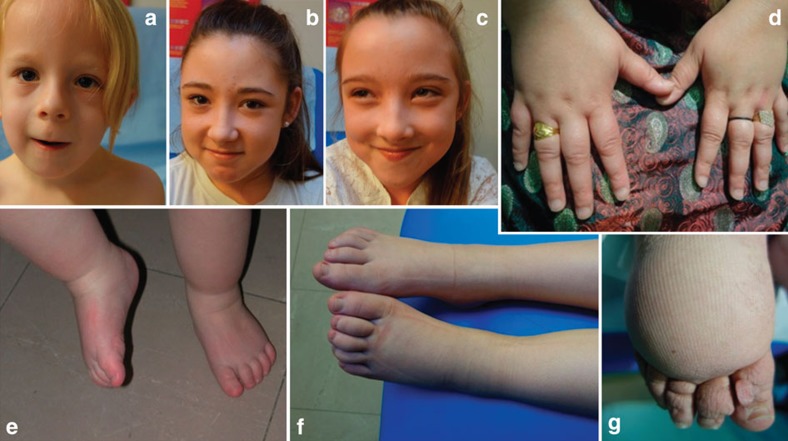
Clinical findings in GLD patients with *PIEZO1* mutations. Faces of (**a**) GLD6:II.1 at age 3.5 years, (**b**) GLD4:II.2 at 14 years and (**c**) GLD4:II.3 at 12 years, all demonstrating epicanthic folds and no current signs of facial swelling. (**d**) Hand swelling in subject GLD1:II.3. Foot swellings in (**e**) GLD4:II.2 at age 3 years and (**f**) GLD4:II.2 at age 14 years, and (**g**) subject GLD1:II.3 at age 34 years.

The phenotype is distinct from Hennekam syndrome as none of our GLD patients had the dysmorphic features associated with HS, nor the learning disabilities or seizures, nor was the swelling severe (Fig. 1).

### Exome sequencing was performed on the three probands

No variants were found in *FAT4*. Applying an autosomal recessive inheritance model requiring at least one previously unobserved rare homozygous, or two rare heterozygous, protein altering variants in the same gene in all three individuals highlighted *PIEZO1* as the only candidate gene matching these criteria. On follow-up with Sanger sequencing, we found the variants co-segregate with the disease status in the families (Supplementary Fig. 1).

### Sanger sequencing

Additional GLD probands (*n=*10) with a similar phenotype were sequenced for all *PIEZO1* coding exons and their associated splice sites. Variants were identified in three of these probands (Table 1), one of which was the nonsense mutation (c.G4888T; p.E1630X) also observed in family GLD1. The variants were assessed in all available relatives, and were found to co-segregate with the GLD phenotype (Supplementary Fig. 1). Two missense variants, c.C2815A and c.C7374G, in GLD6:II.1 were inherited from the mother (GLD6:I.2) and at present it is unclear which is responsible for the disease phenotype. In total, we identified 10 *PIEZO1* variants in 6 families (Supplementary Fig. 2). The 2 variants in *cis* in GLD6:II.1 have been observed (rs201226914 and rs202127176, respectively) with a minor allele frequency of 0.0002, however, none of the rest were present in dbSNP, or have been identified by the 1000 Genomes Project or observed in a cohort of 900 control samples.

### Variant analysis suggests they are pathogenic

**Figure 2:**
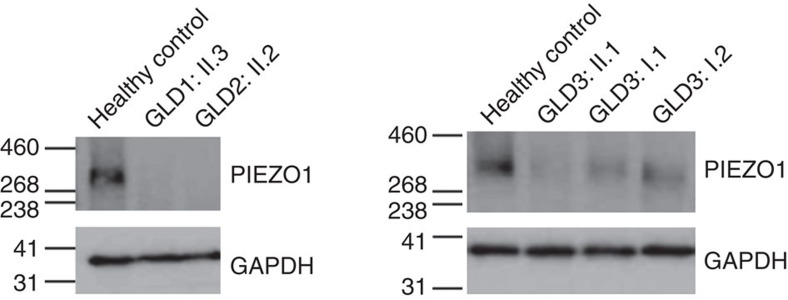
PIEZO1 protein expression is defective in GLD patients. Western blot analysis of PIEZO1 protein expression isolated from RBCs membranes of a healthy control subject and GLD patients. A second gel was run in parallel using GAPDH as loading control. The position of molecular mass markers (in kDa) is indicated to the left of the gel. GLD1:II.3, homozygous nonsense mutation p.E1630X; GLD2:II.2, compound heterozygous nonsense mutations p.E755X/p.Q2228X; GLD3:II.1, compound heterozygous splice site c.3796+1G>A and missense p.V2171F mutations; GLD3:I.1, heterozygous splice site c.3796+1G>A mutation; GLD3:I.2, heterozygous missense p.V2171F mutation.

The five missense mutations alter evolutionarily conserved amino acid residues (Supplementary Fig. 3) and are predicted to have a damaging effect on protein function according to MutationTaster^6^; all are located within the C-terminal domain of the molecule except p.L939M (Supplementary Fig. 2). The two splice-site mutations are predicted in HSF^7^ and MutationTaster to have significant impact on the splicing of the transcript. Analysis of complementary DNA (cDNA) from various family members of GLD3 and GLD4 showed two fragments of differing size, indicating splicing is being affected. The c.3796+1G>A variant in GLD3 causes skipping of exon 26 (Supplementary Fig. 4), and the c.1669+1G>A variant in GLD4 leads to the inclusion of intron 13–14 (Supplementary Fig. 5). Western blot results of the c.3796+1G>A variant in GLD3 showed a reduction in PIEZO1 expression levels (Fig. 2, Supplementary Fig. 6). The three nonsense mutations are predicted to lead to premature termination of the protein. No truncated PIEZO1 protein products were identified in western blot analysis in GLD1:II.3 and GLD2:II.2 (Fig. 2, Supplementary Fig. 6), suggesting that the truncated protein is not stable and therefore degraded.

### Blood results show mild abnormalities

**Figure 3:**
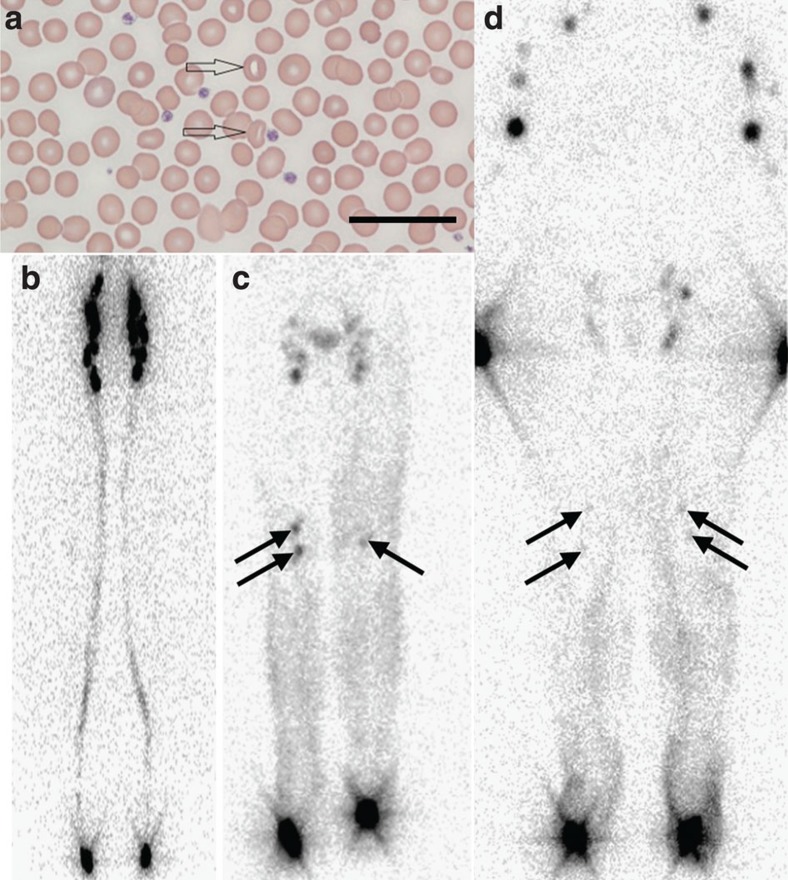
Blood analysis and lymphoscintigraphy. (**a**) Blood film showing occasional stomatocytes (arrows) in GLD1:II.2. Scale bar, 20 μm. (**b**) Lower limb lymphoscintigraphy in an unaffected subject showing symmetrical migration of radionuclide through discrete lymph vessels 2 h after injection. (**c**) Lower limb lymphoscintigraphy of GLD4:II.3 and (**d**) four limb lymphoscintigraphy in GLD1:II.3. The three lymph scans have been aligned so that injection sites in the feet are all at the bottom of the panel with the groin area all at the same level at the top of **b** and **c**. The injection sites in the hands in **d** are the dark areas on either side of the groin region and axillary lymph nodes are visible at the top of **d**. The lymph scans of the two patients (**c**,**d**) show distinctive changes with poor uptake of tracer in the groin and axillae at 2 h, with evidence of rerouting in the lower limbs (seen as the dark shading of the lower limb). Popliteal lymph nodes (arrows) show prominent uptake of tracer, which is unusual and represents deep rerouting of the interstitial fluid.

Heterozygous variants in *PIEZO1* are associated with DHS. Careful inspection of blood films from our affected individuals demonstrates occasional stomatocytes and spherocytes (Fig. 3a, Supplementary Fig. 7). The red cell abnormalities are subtle but convincing. Even fewer stomatocytes are also observed in carriers (for example, GLD5:I.2 heterozygous for p.E1630X, Supplementary Fig. 7a). It is also noticeable that the two affected individuals in GLD4 show quite marked spherocytosis (Supplementary Fig. 7c,d) but the blood film from their carrier mother was unremarkable with only occasional spherocytes (Supplementary Fig. 7b). All were asymptomatic, and blood test results are summarized in Supplementary Table 1.

### Lymphoscintigraphy

Four of the patients have had lymphoscintigraphy, two of all four limbs and two of the lower limbs. The lymph scan images show striking consistency and symmetry (Fig. 3). In the lower limbs, all the patients have deep rerouting as demonstrated by the popliteal lymph node uptake^8^. There is also superficial rerouting through the skin. The rerouting suggests failure of superficial lymphatic collector function.

## Discussion

PIEZO1 is a calcium permeable mechanically activated ion channel^9^,^10^ and is found in the plasma membrane of various cell types. Mechanotransduction is important for many physiological processes in the body and is also vital for regulation of embryonic development. In mouse, Piezo1 mRNA abundance increased gradually from E9.5 to E15.5 and was sustained through to birth^11^.

A haematologic phenotype has been observed in morpholino knockdown of *piezo1* in zebrafish^12^. Various mouse models are embryonic lethal when Piezo1 is completely inactivated, but the heterozygous mice are viable and fertile^13^,^14^. Vascular remodelling was less organized and with a significantly lower number of major vessels, indicating that Piezo1 has an important role during the development of the blood vasculature. There is also evidence that PIEZO1 has a role during the development of the lymphatic vasculature, as it was observed in lymphatic vessels of the peritoneum in human foetal tissue at 17 weeks of gestation^11^.

*PIEZO1* is already associated with another human disease; DHS with or without pseudohyperkalaemia and/or perinatal oedema (OMIM #194380)^15^. DHS is an autosomal dominant, pleiotropic syndrome with significant inter and intra familial variation^11^,^16^,^17^. DHS patients typically present with mild to moderate haemolytic anaemia characterized by increased cation leak from red cells, causing erythrocyte dehydration. Pseudohyperkalaemia occurs as potassium leaks from the red cells. Splenomegaly, jaundice and NIHF variably occur, the NIHF being transient and not related to anaemia^18^. The majority of DHS variants described in *PIEZO1* is missense and predicted as gain-of-function^11^, leading to altered channel kinetics. This could explain the increased permeability of erythrocyte membranes to cations, causing the clinical presentation of haemolysis in DHS patients^17^,^19^.

Typically, haematology results for DHS patients show evidence of mild to moderate haemolysis, with normal or reduced haemoglobin and reticulocytosis, with variable numbers of stomatocytes seen on the blood film^16^. Hyperkalaemia is often recorded if there is a significant delay between venesection and analysis. On close inspection, haematological analysis in our GLD cases show evidence of a mild, asymptomatic haemolytic anaemia with more subtle changes than that seen in DHS, and with no documented pseudohyperkalaemia, although the number of cases is too small to clearly link genotype and phenotype. It is not clear why red cells adopt a stomatocytic shape in conjunction with increased cation leak, and the spherocytes seen in one family (GLD4) are likely to also be due to the *PIEZO1* mutation, and suggest that some cases of spherocytosis could be caused by mutations in this gene.

Disorders of the lymphatic system have an important role in the cause of NIHF, and 15% of NIHF cases are reported to be due to lymphatic dysplasia^20^. The NIHF in our GLD cases is clearly related to a lymphatic phenotype, many of the neonates had evidence of chylothoraces and presented in childhood with lymphoedema and an abnormal lymphoscintigraphy. The hydrops in the DHS cases are reported to be unrelated to anaemia^18^ and therefore not of a haematologic origin. Instead, we suspect it is highly likely to be lymphatic in origin and a case with severe generalized oedema and cystic hygromas, suggesting a lymphatic origin has been reported^21^.

Interestingly, the variants identified in this cohort cause a mild form of DHS in affected individuals and carriers but biallelic variants have a high incidence of NIHF (7 of the 10 affected cases). This may be lethal in the perinatal period (*n=*2). Survivors may present later with lymphoedema of the peripheries (mainly lower limbs but also arms (*n=*4), face (*n=*3) and genitalia (*n=*1)), with or without chylothoraces (*n=*2) and intestinal lymphangiectasia (*n=*1). The recurrence of lymphatic problems in childhood observed in our GLD patients is comparable to that seen in Turner syndrome^22^. Four patients of our cohort had severe, recurrent facial cellulitis with significant morbidity (high pyrexia and frequent admission to intensive care).

The high incidence of perinatal oedema in both DHS and GLD cannot be ignored. Monoallelic variants, presenting with DHS in a parent but perinatal oedema in the offspring, are not fully understood^23^,^24^, but possible explanations include an unidentified variant on the other allele, modifier genes or stochastic events. DHS patients with biallelic missense mutations have been reported in one family^15^. They presented with a more severe haemolytic phenotype compared with heterozygous family members, but no pseudohyperkalaemia or perinatal oedema was observed in that particular family. The literature so far has not described a lymphatic phenotype in the patients who survived the perinatal oedema or patients with DHS but this requires further investigation. This could possibly be explained by the lack of long term follow-up or ascertainment bias.

Too little is known about PIEZO1 to say whether different forms of the protein exist for certain key mechanisms or tissues, thus leading to the DHS and GLD phenotypes. The effects of these *PIEZO1* mutations are significantly more severe prenatally, with complete resolution of the oedema after birth in some cases. This must reflect on the specific role of PIEZO1 on the lymphatics. The lymphatics undergo a significant transformation at birth under the effect of glucocorticoids^25^. Perhaps PIEZO1 is required to maintain the lymphatics in the foetal state but not required to such a degree after birth. More investigations are needed to fully understand the role of PIEZO1 in GLD, as well as DHS.

In summary, we have identified *PIEZO1* mutations that cause an autosomal recessive form of GLD associated with NIHF and chronic peripheral primary lymphoedema. Together with *CCBE1* and *FAT4*, this is the third gene to be associated with GLD. The observation of lymphoedema provides evidence of a role for PIEZO1 in the development of lymphatic structures. It remains to be elucidated how far the established ion channel function of PIEZO1 can account for the different phenotypical aspects of DHS and GLD and whether at least some defects are the consequences of a different role of PIEZO1 during development.

## Methods

### Patient ascertainment

GLD probands with no identifiable mutations in *CCBE1* were selected for study. Ethical approval for this study was obtained from the South West London Research Ethics Committee (REC Ref: 05/Q0803/257). Probands and their families were ascertained through the Primary Lymphoedema Clinic at St George’s Hospital, London, UK and written informed consent was obtained from all participants. All affected individuals and their family members underwent a detailed clinical examination.

### Targeted capture and massive parallel sequencing

For GLD1, sequencing libraries were made following the protocol from Roche/Nimblegen's SeqCap EZ Exome Library v2.0 kit (Roche NimbleGen, Inc). The libraries were then sequenced on a HiSeq2000 (Illumina), with paired end, 101-bp reads. For GLD2 and GLD3, whole exome capture was performed using the SureSelect Target Enrichment System (Agilent). This was followed by sequencing on a HiSeq2000 (Illumina) with 100-bp paired end reads. Sequence reads for all families were aligned to the reference genome (hg19) using Novoalign (Novocraft Technologies SdnBhd). Duplicate reads, resulting from PCR clonality or optical duplicates and reads mapping to multiple locations were excluded from downstream analysis. Depth and breadth of sequence coverage were calculated with custom scripts and the BedTools package^26^.

### Variant analysis

All exomes from the three families were passed through the same variant analysis pipeline. Single-nucleotide substitutions and small indel variants were identified and quality filtered within the SamTools software package^27^ and in-house software tools^28^. Variants were annotated with respect to genes and transcripts with the Annovar tool^29^. Variants were filtered for novelty by comparing them with dbSNP135 and 1000 Genomes SNP calls and with variants identified in 900 control exomes (primarily of European origin), which were sequenced and analysed by the method described above for GLD2 and GLD3. Summary statistics for the exome sequencing is given in Supplementary Tables 2 and 3. First the exomes were checked for variants in *FAT4*, as none were found the analysis of the exome-variant profiles was then performed under a model of a rare autosomal recessive disorder; this model required one previously unobserved homozygous variant for the consanguineous GLD1 or two previously unobserved heterozygous variants in trans in the same gene for all affected individuals in GLD2 and GLD3.

### Confirmation sequencing

Samples of available family members were analysed by Sanger sequencing for the variants found in their respective proband. Further 10 samples with a GLD phenotype were analysed for sequence variants in all of *PIEZO1*, hence primers were designed for the coding regions and associated splice sites of *PIEZO1* using Primer3 software^30^ (primer sequences in Supplementary Table 4). PCR products were sequenced using BigDye Terminator v3.1 chemistry (Life Technologies) and an ABI3130xla Genetic Analyzer (Life Technologies). Sequencing traces were visually inspected in Finch TV v1.4 (Geospiza Inc, Seattle, WA, USA) and aligned to a wild-type reference using CLC Sequence Viewer (CLC bio-Qiagen, Denmark). Identified variants were checked in dbSNP (http://www.ncbi.nlm.nih.gov/SNP/), 1000 Genomes Project (http://www.1000genomes.org/) and in a cohort of 900 control samples for novelty. The variants were also analysed in either Mutation Taster^6^ or HSF^7^ for genetic effect.

### RNA/cDNA analysis of splice variants

Blood was collected in PAXgene Blood RNA tubes and RNA purified using PAXgene Blood RNA Kit (PreAnalytix). About 500 ng of total RNA was used as template for cDNA generation using RT Superscript II (Invitrogen). Oligos used for splice variant amplification in family GLD3 were exon 26F 5′-AACCTCATCAGCGACTTTCTC3-3′ and exon 26R 5′-ATACGCTCCATCTGTCTTTTC-3′; and in family GLD4 were exon 13F 5′-TGGAGCATCACCTACCACAG-3′ and exon 13R 5′-GGCTGTAGTAGACCTGGAAGA-3′.

### Western blot

RBCs were isolated from blood and lysed by three cycles of freezing and thawing in 5 mM KH_2_PO_4_ containing protease inhibitors. Lysate was centrifuged at 16,000*g* for 1 h and membrane fraction was washed three times in 5 mM KH_2_PO_4_ containing proteases inhibitors. Membrane fraction was resuspended in RIPA buffer (20 mM Tris HCl pH 7.5, 150 mM NaCl, 1% Igepal CA-630, 0.5% sodium deoxycholate and 0.1% SDS) and centrifuged at 16,000*g* for 1 h. Supernatant containing membrane proteins was prepared in urea buffer (200 mM Tris HCl pH 6.8, 5% SDS, 8 M urea, 100 mM DTT and 0.1% bromophenol blue). Samples were heated for 10 min at 37 ^o^C and loaded in NuPAGE Novex 3-8 % Tris-Acetate gels with NuPAGE Tris-Acetate SDS running buffer (Life technologies). Proteins were transferred on a polyvinylidene difluoride membrane, and PIEZO1 protein was detected with a specific antibody (15939-1-AP, Proteintech). GAPDH was used as a loading control and detected with a specific antibody (ab8245-100, Abcam). See Supplementary Fig. 6 showing the uncropped western blots.

### Haematology and biochemistry analysis

Available family members and/or probands of four of the six families had blood analysis including tests for Hb, reticulocytes, potassium, lactate dehydrogenase and haptoglobin. Blood films were prepared with May–Grünwald–Giemsa stain and examined using light microscopy, looking specifically for red cell abnormalities including stomatocytes. Each film was examined by two experienced haematologists. Where possible, we looked at haematology reports in patient notes for the other families and checked for evidence of haemolysis.

### Lymphoscintigraphy

Lymphoscintigraphy is the imaging of the lymphatic system by injecting radioactive isotope (technetium-99m) into the web spaces between the toes and/or fingers and quantification of uptake into the inguinal lymph nodes for foot injections and axillary nodes for hand injection after 2 h with a gamma camera.

## Additional information

**Accession number:** All the samples and the variants presented in Table 1 have been submitted to the Leiden Open Variation Database(http://databases.lovd.nl/shared/genes/PIEZO1) with the following accession IDs: Patients from GLD1 as Individual IDs: 43814 and 43815; patients from GLD2 as Individual IDs: 43816 and 43817; GLD3 as Individual ID: 43818; GLD4 as Individual IDs: 43819 and 43820; GLD5 as Individual ID: 43821; and GLD6 as Individual ID: 43823.

**How to cite this article:** Fotiou, E. *et al*. Novel mutations in *PIEZO1* cause an autosomal recessive generalized lymphatic dysplasia with non-immune hydrops fetalis. *Nat. Commun.* 6:8085 doi: 10.1038/ncomms9085 (2015).

## Supplementary Material

Supplementary Information Supplementary Figures 1-7, Supplementary Tables 1-4 and Supplementary References

## Figures and Tables

**Table 1:** Clinical and genetic findings in GLD patients with *PIEZO1* mutations.

				**Genotyping**			**Antenatalhistory**		**Neonatalhistory**	**Lymphoedema(postnatal andonwards)**			**Additionalclinicalfeatures**	
	**ID**	**Gender**	**Age**	**Nucleotidevariant**	**Exon**	**Proteinalteration**	**NIHF**	**PH**	**Oedema**	**Onset**	**Limbs**	**Face**	**Dysmorphicfeatures**	**Othercomments**
GLD1	II.2*	M	26	c.G4888T†	36	p.E1630X	—	—	—	6 years	4 limbs	Y	Micrognathia	Recurrentfacialcellulitis,DVT,genitaloedema
	II.3	F	34	c.G4888T†	36	p.E1630X	N	N	N	9 years	4 limbs	(Y)	N	CT and Pl Eat age 2 years,recurrentcellulitisin face andLL, varicoseveins
GLD2	II.1	M	NA	c.G2263Tc.C6682T	1746	p.E755Xp.Q2228X	Pl E,A, S	Y	NA	NA	NA	NA	NA	Died *in utero*at 34 weeks,amyoplasiaof diaphragm
	II.2*	M	9	c.G2263Tc.C6682T	1746	p.E755Xp.Q2228X	Pl E	Y	Mildgeneralizedoedemawith Pl E	At birth	4 limbs	(Y)	Periorbitaloedema,cuppedsimpleears,epicanthicfolds,micrognathia	ASD, GR,CT, shortstature,pectusexcavatum,genitaloedema,splenomegaly
GLD3	II.1*	F	16	c.3796+1G>A c.G6511T	26i45	Donor splicesite p.V2171F	Pl E, A	—	Y (but resolvedrapidly)	6 years	(4 limbs)L LL	(Y)	N	GR, intestinallymphangiectasia,granulomaannularescoliosis.Oedemaimproved onlow fat diet
GLD4	II.1	F	NA	No DNAavailable	—	—	Pl E,severe	—	Generalizedoedema	At birth	4 limbs	Y	N	Diedat age4 weeks
	II.2	F	14	c.1669+1G>A c.C7289T	13i50	Donorsplicesitep.P2430L	Pl E	Y	L footpedal	L LL atbirth, RLL 10 years	B LL	(Y)	Epicanthicfolds	Cellulitis × 3
	II.3	F	12	c.1669+1G>A c.C7289T	13i50	Donorsplicesitep.P2430L	Mild	—	Oedemaresolvedbon day 1	R LL4y L LL8 years	B LL	(Y)	Epicanthicfolds	Cellulitis ×2 LLCellulitis ×1 face
GLD5	II.2	M	3.5	c.G4888T†	36	p.E1630X	N	Y	Head andneckswelling,hydroceles	At birth	(4 limbs)	Y	N	Intermittentfacialcellulitis,bilateralsensorineuraldeafness,hypothyroidism,milddevelopmentaldelay
GLD6	II.1	F	3.5	c.C7366T c.C7374G/c.C2815A	51 51/21	p.R2456Cp.F2458L/p.L939M	Pl E, A, S	Y	Generalizedoedema atbirth, CT	None	NA	NA	Webbed neck,periorbitaloedema,‘prune’belly	GR,hypothyroidat birth

A, ascites; ASD, atrial septal defect; B, bilateral; CT, chylothorax/chylothoraces; DVT, deep vein thrombosis; F, female; GR, gastro-esophageal reflux; L, left; LL, lower limb, M, male; N, no; NIHF, non-immune hydrops fetalis; PH, polyhydramnios; Pl E, bilateral pleural effusions; R, right; S, skin oedema; Y, yes; —, not available; NA, not applicable.

Brackets indicate that swellings have been recorded for that segment in the medical notes, but are currently resolved.

^*^Exome sequenced.

^†^homozygous variant.
