# Author Correction: Novel mutations in *PIEZO1* cause an autosomal recessive generalized lymphatic dysplasia with non-immune hydrops fetalis

**DOI:** 10.1038/s41467-019-09905-4

**Published:** 2019-04-26

**Authors:** Elisavet Fotiou, Silvia Martin-Almedina, Michael A. Simpson, Shin Lin, Kristiana Gordon, Glen Brice, Giles Atton, Iona Jeffery, David C. Rees, Cyril Mignot, Julie Vogt, Tessa Homfray, Michael P. Snyder, Stanley G. Rockson, Steve Jeffery, Peter S. Mortimer, Sahar Mansour, Pia Ostergaard

**Affiliations:** 1 Cardiovascular and Cell Sciences Institute, St. George’s University of London, Cranmer Terrace, London, SW17 0RE UK; 2 Department of Medical and Molecular Genetics, Division of Genetics and Molecular Medicine, Kings College London School of Medicine, Guy’s Hospital, London, SE1 9RY UK; 3 Division of Cardiovascular Medicine, Stanford University, Stanford, California 94305 USA; 4 Department of Genetics, Stanford University, Stanford, California 94305 USA; 5 Department of Dermatology, St. George’s Healthcare NHS Trust, London, SW17 0QT UK; 6 South West Thames Regional Genetics Unit, St. George’s University of London, London, SW17 0RE UK; 7 Pathology Department, St. George’s University of London, London, SW17 0RE UK; 8 Department of Haematological Medicine, King’s College London School of Medicine, King’s College Hospital, London, SE5 9RS UK; 9 Département de Génétique, APHP, GH Pitié-Salpêtrière, Centre de Référence des Déficiences Intellectuelles de Causes Rares, 75013 Paris, France; 10 West Midlands Regional Genetics Service, Clinical Genetics Unit, Birmingham Women’s Hospital, Birmingham, B15 2TG UK

Correction to: *Nature Communications* 10.1038/ncomms9085, published online 03 September 2015.

This Article contains an error in the last sentence of the ‘Variant analysis suggests they are pathogenic’ section of the Results, which incorrectly reads ‘No truncated PIEZO1 protein products were identified in western blot analysis in GLD1:II.3 and GLD2:II.2 (Fig. 2, Supplementary Fig. 6), suggesting that the truncated protein is not stable and therefore degraded’. This should read ‘No full-size PIEZO1 protein products were identified in western blot analysis in GLD1:II.3 and GLD2:II.2 (Fig. 2, Supplementary Fig. 6); the three nonsense mutations are predicted to lead to premature termination of the protein, hence it is possible that those truncated proteins will be non-functional or even unstable and degraded’. The error has not been fixed in the PDF or HTML versions of the Article.

