# Supplementary material for: Novel mutations in PIEZO1 cause an autosomal recessive generalized lymphatic dysplasia with non-immune hydrops fetalis
Source: Nat Commun. 2015 Sep 3;6:8085. doi: 10.1038/ncomms9085 (PMC4568316; doi:10.1038/ncomms9085)
Supplement: Supplementary Information — Supplementary Figures 1-7, Supplementary Tables 1-4 and Supplementary References [file ncomms9085-s1.pdf]

Supplementary Fig. 1: Pedigree structure and mutation status of the families included in this study

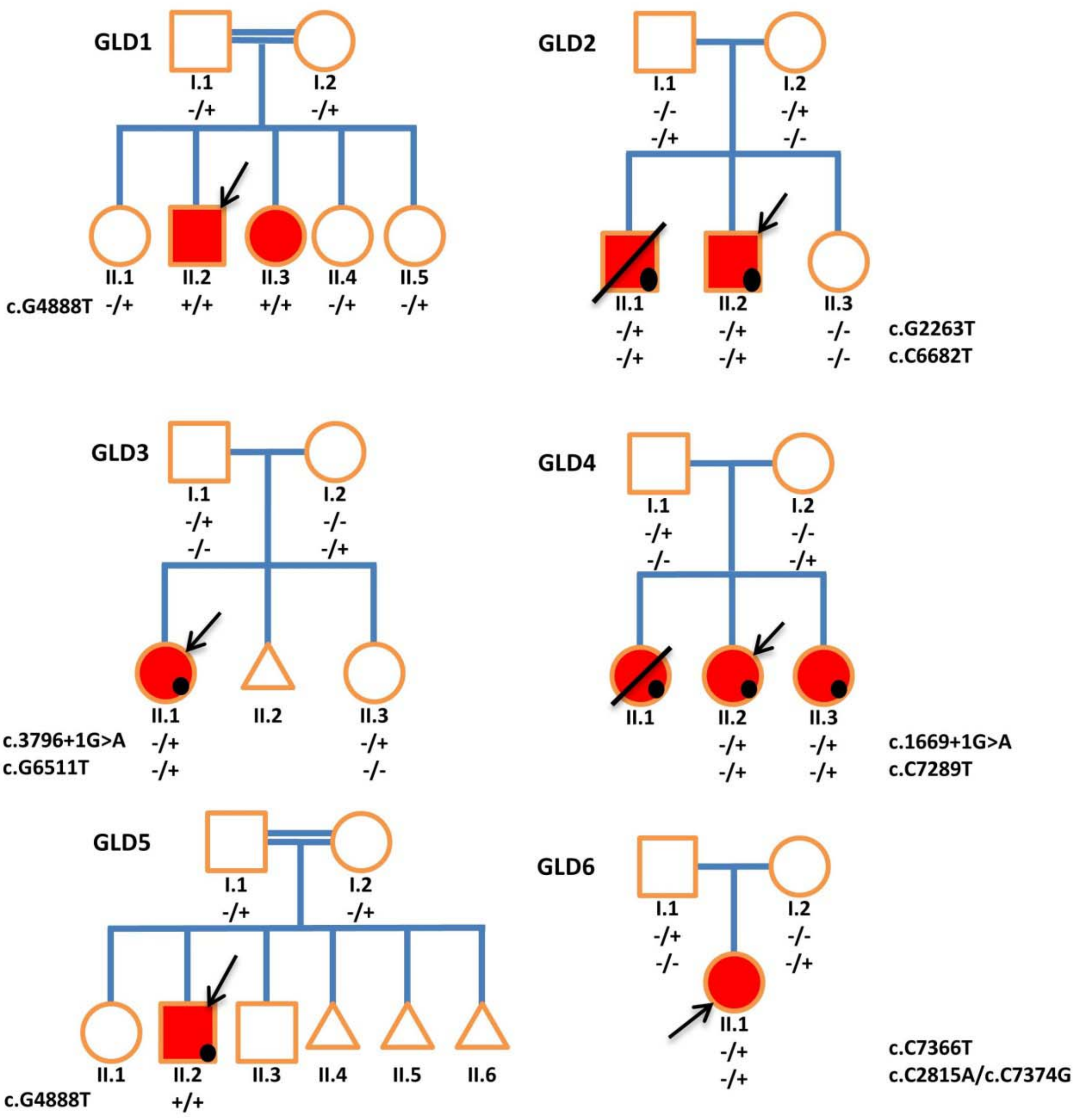

GLD affected individuals are indicated with filled circles or squares and black dots indicate if the patients also presented with edema or hydrops fetalis *in utero*. *PIEZO1* genotypes are indicated for individuals where DNA was available for research; +: mutated allele, -: normal allele. Arrows indicate proband. Probands from GLD1, GLD2 and GLD3 were selected for exome sequencing analysis. GLD6:II.1 has coinherited two variants in *cis* from the mother.

Supplementary Fig. 2: Location of the identified mutations in *PIEZO1*

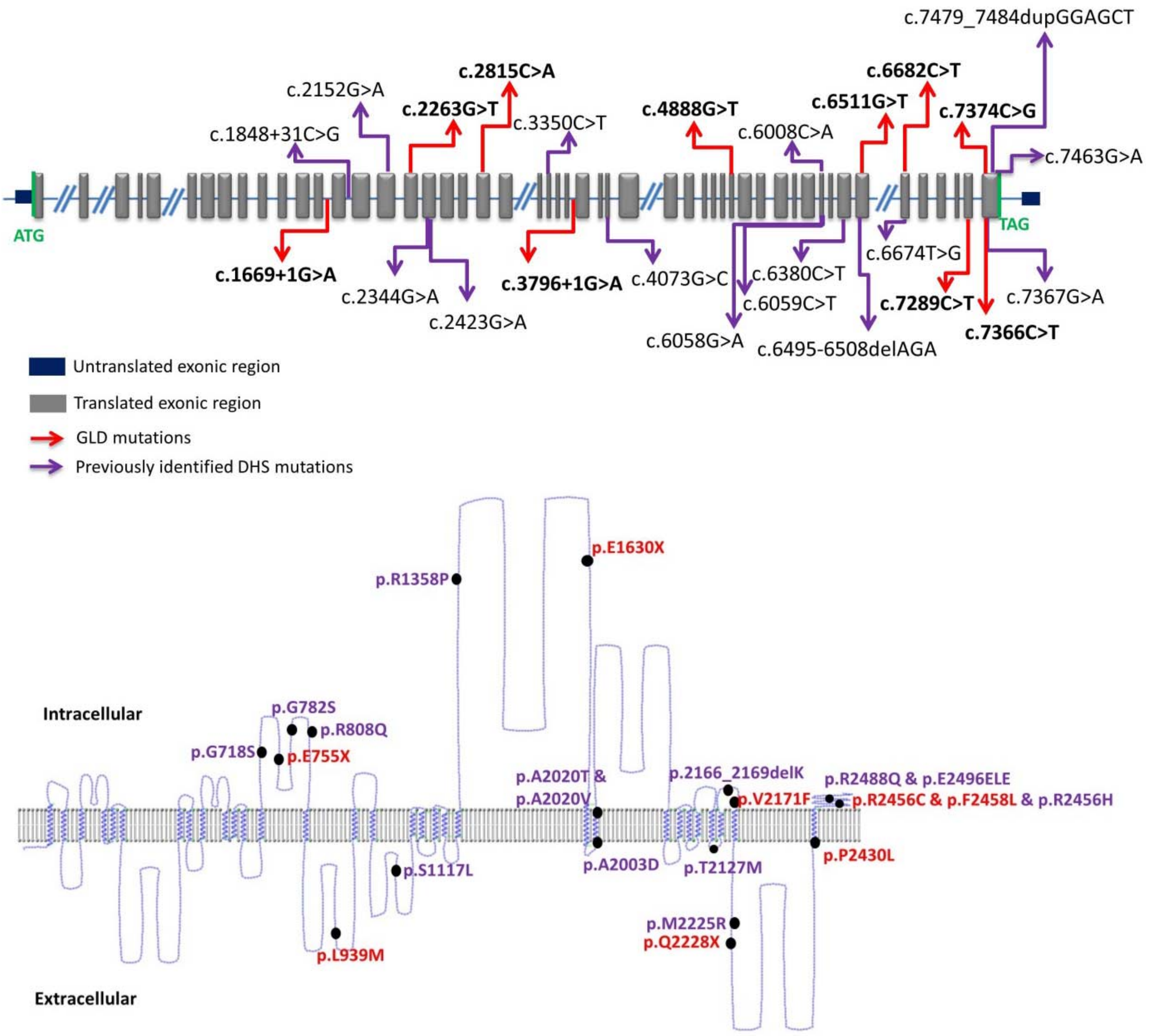

The top panel shows the genomic organization of *PIEZO1* and the position of the identified mutations in relation to exons. Variants identified in the GLD families are in bold and with red arrows, variants from publications on DHS are in non-bold with purple arrows[1-5].

The bottom panel shows the predicted transmembrane regions of human PIEZO1 using TMRPres2D software[6]. The black dots indicate the approximate location of the mutations (excluding splice variants). The variants in red are from the six GLD families and the variants in purple are from the DHS publications.

Supplementary Fig. 3: Conservation of the residues altered by the *PIEZO1* mutations.

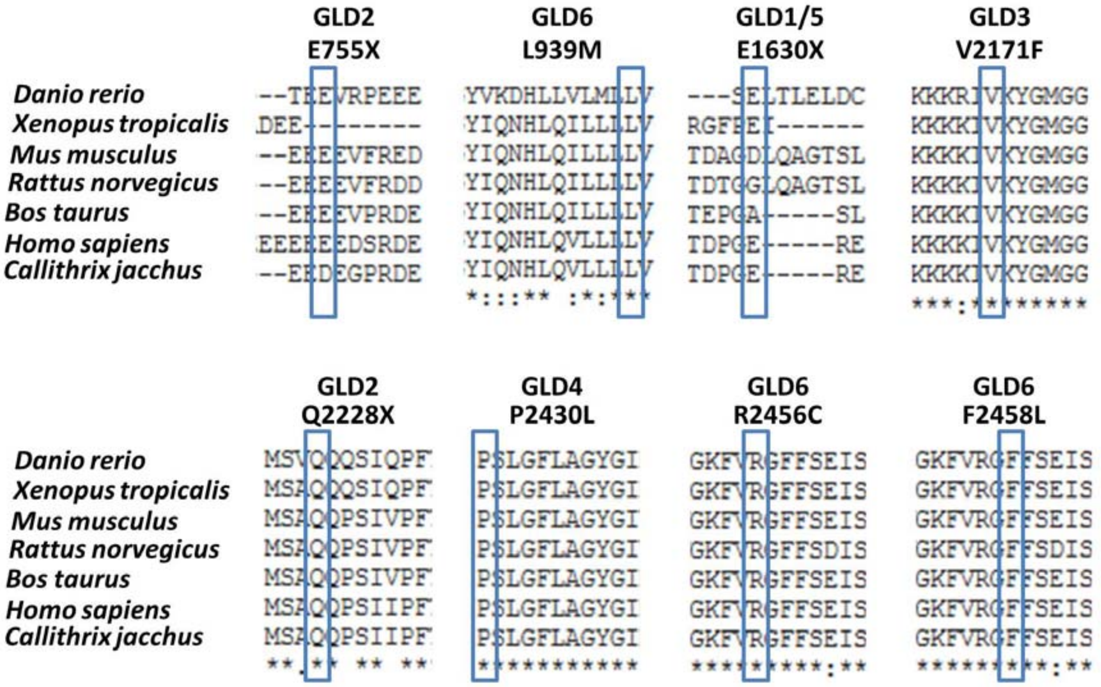

The protein sequences of *Danio rerio* (AC#XP_696355.4), *Xenopus tropicalis* (AC#XP_002933721.2), *Mus musculus* (AC#ADN28064.1), *Rattus norvegicus* (AC#NP_001070668.2), *Bos taurus* (AC#XP_010813024.1), *Homo sapiens* (AC#AGH27891.1) and *Callithrix jacchus* (AC#XP_008984619.1) PIEZO1 were aligned with Clustal Omega (www.clustal.org/omega/*)*. Asterisks denote highly conserved residues between the different species.

Supplementary Fig. 4: Analysis of splicing variants in GLD3 and GLD4

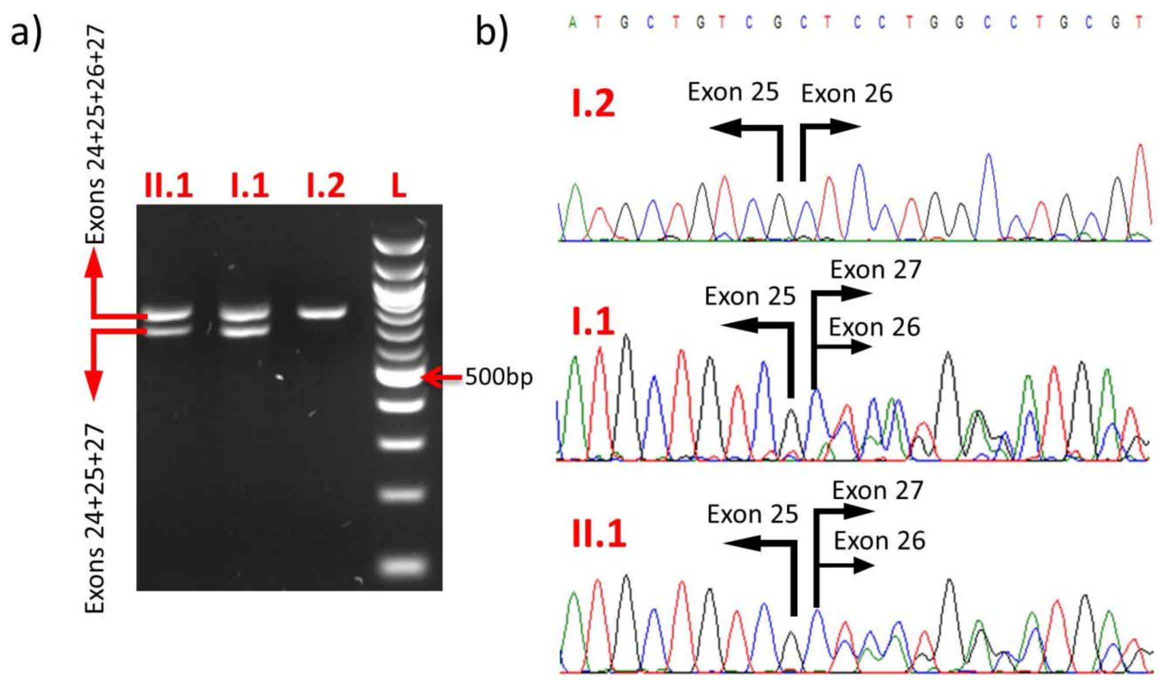

a) A 2% agarose gel electrophoresis (SYBR Safe stained) of PCR products amplified from cDNA in the patient (II.1), the father (I.1) and the mother (I.2) of family GLD3. A lane of 100bp molecular weight marker (L) is also shown (arrow at 500bp). The primers were located across exon-exon boundaries at exons 23-24 and 28-29 to capture the effect of the splice variant, c.3796+1G>A. The shorter amplicon indicated a deletion of 97 nucleotides. **b)** The PCR products were Sanger sequenced to further establish the nature of the splicing and confirms skipping of exon 26. The arrows indicate the exon-exon boundaries. Both II.1 and I.1 have clear sequencing traces until the exon 25-26 boundary, after which point both have exon 27 sequence running on top of the exon 26 indicating that exon 26 has been skipped on one of the alleles.

Supplementary Fig. 5: Analysis of splicing variants in GLD4

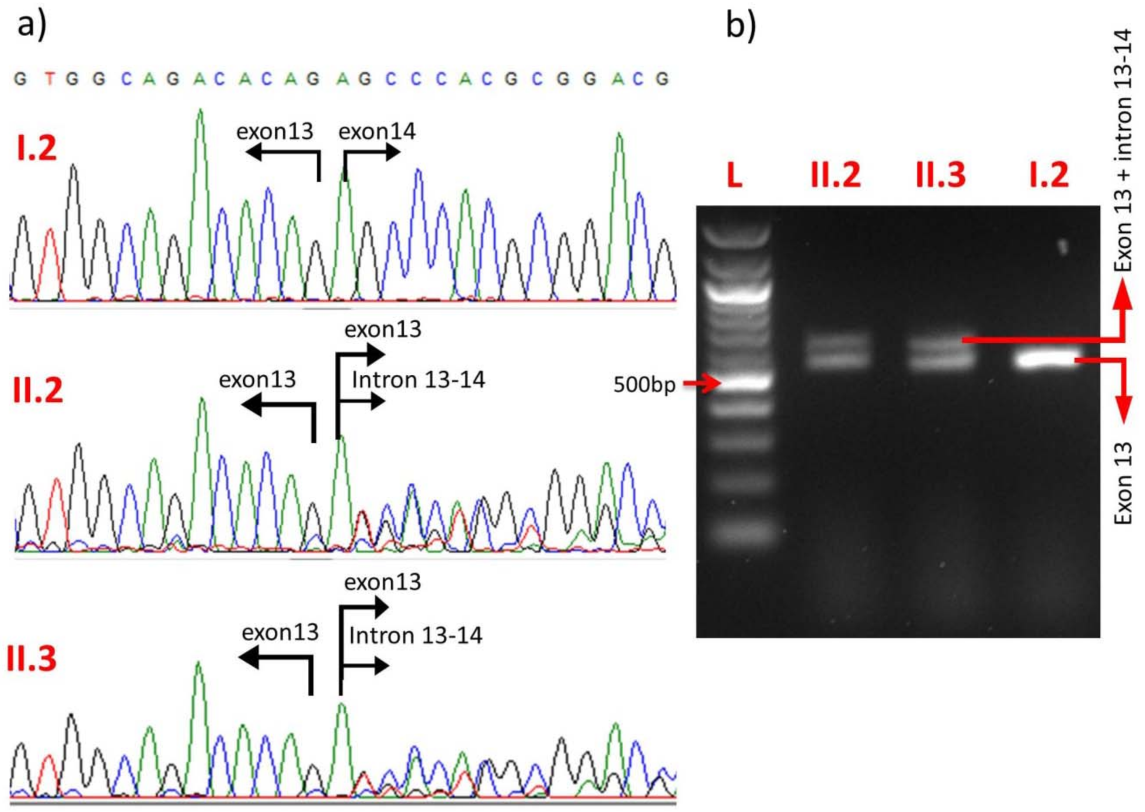

a) Sanger sequencing of cDNA from family GLD4. Sequencing trace (I.2) is from the non-carrier mother and traces (II.2 and III.3) are from the daughters who both carry the splice variant c.1669+1G>A. The arrows indicate the exon-exon boundary. Both II.2 and III.3 have clear sequencing traces until the boundary between exons 13 and 14, after which point both have intron 13-14 running on top of exon 14 indicating that intron 13-14 has not been spliced out. b**)** A 2% agarose gel (SYBR Safe stained) of the PCR products from cDNA in GLD4. The longer amplicon indicated the retention of 81 nucleotides. A lane of 100bp molecular weight marker (L) is also shown (arrow at 500bp).

Supplementary Fig. 6: PIEZO1 protein expression is defective in GLD patients

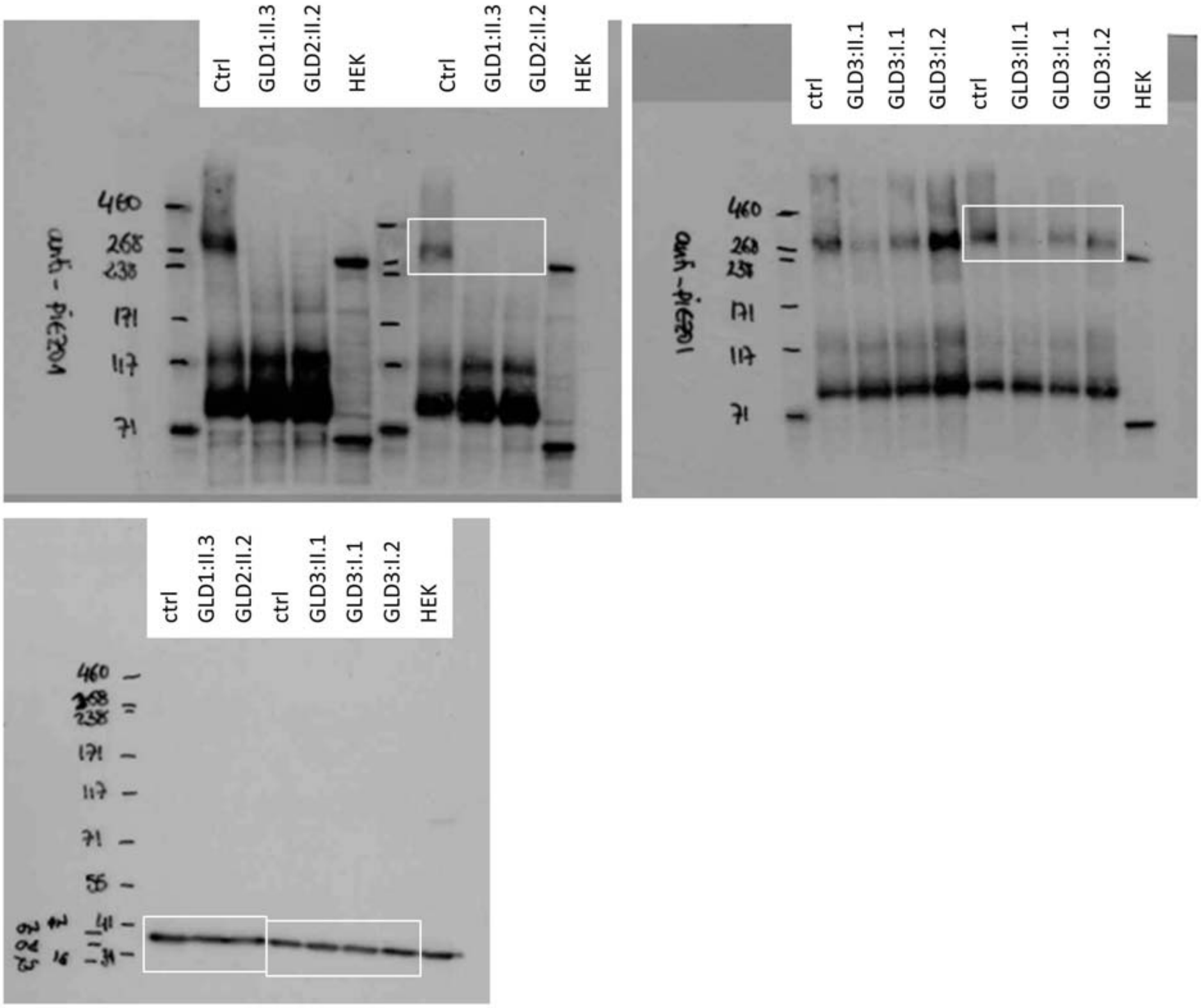

Original western blots (one representative of two experiments is shown) with white outline indicating which sections have been used in Fig. 2 in the paper. The two blots in the top panel are PIEZO1 and the blot in the bottom panel is the control blot with GAPDH. See legend to Fig. 2 for interpretation.

Supplemental Figure 7. Blood films from affected individuals and carriers

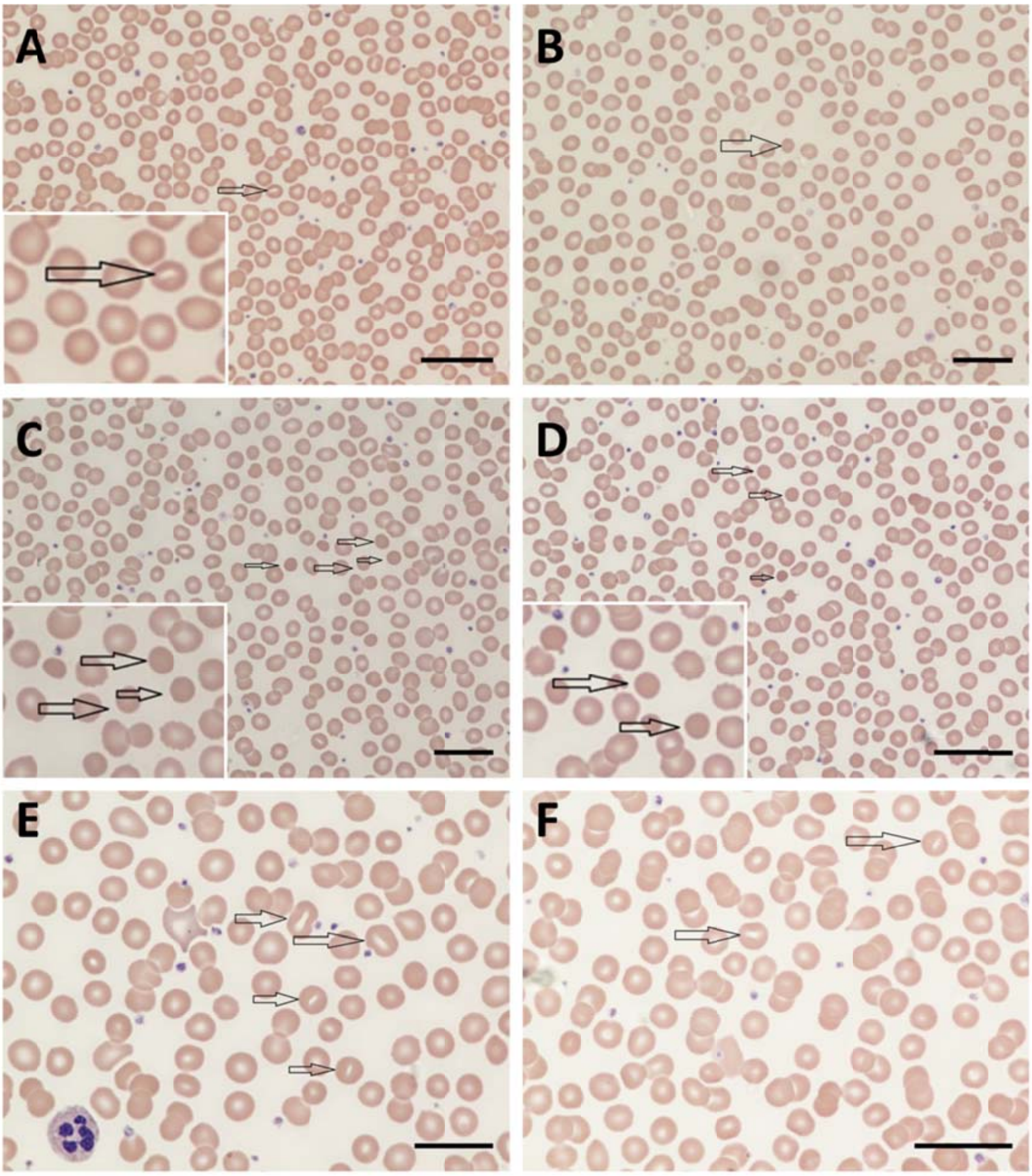

Findings of interest are highlighted with arrows. **A.** GLD5:I.2 carrier of c.G4888**T**/p.E1630X mutation has very occasional stomatocytes. Insert: zoom in on stomatocyte; **B.** GLD4:I.2 carrier of c.1669+1G>A has very occasional spherocytes; **C.** GLD4:II.2 and **D.** GLD4:II.3 both compound heterozygous for c.1669+1G>A and c.C7289T/p.P2430L have spherocytes. Inserts: zoom in on spherocytes; **E.** GLD6:I.2 carrier of c.C2815A/p.L939M and c.C7374G/p.F2458L in *cis* has occasional stomatocytes; **F.** GLD6:I.1 carrier of c.C7366T/p.R2456C occasional stomatocytes. All scale bars indicate 20µm,

Supplementary Table 1. Blood results

Ped	Id	Age	Gen-der	Mutation		Hb	Reticulocytes	MCHC	Potassium at 3 hours	Potassium >12hrs	LDH	Hapto-globin	Blood film
						F:(120-160g L^{-1})<br>M:(130-180g L^{-1})	(25-100 x10^9 L^{-1})	(320-356g L^{-1})	(3.5-5.3mmol L^{-1})		(0-175U L^{-1})	(0.4-1.6g L^{-1})<br>(0.5-2.0g L^{-1})	
**GLD1**	**II.2**	26	M	Hom	p.E1630X	125	103	301	4.4	4.6	185	2.02	occasional stomatocytes
**GLD1**	**II.3**	34	F	Hom	p.E1630X	117	N/A	306	3.8	N/A	N/A	N/A	N/A
**GLD4**	**I.2**	35	F	Het	c.1669+1G>A	129	64	338	4	4.6	170	0.64	very occasional spherocytes
**GLD4**	**II.2**	14	F	c-Het	c.1669+1G>A & p.P2430L	131	66	311	4.6	4.7	234	0.89	red cell fragments, spherocytes
**GLD4**	**II.3**	12	F	c-Het	c.1669+1G>A & p.P2430L	116	87	315	3.8	4.5	245	0.15	red cell fragments, spherocytes
**GLD5**	**I.1**	37	M	Het	p.E1630X	142	58	330	4	3.8	151	1.06	unremarkable
**GLD5**	**I.2**	34	F	Het	p.E1630X	129	59	325	4.1	4.6	208	1.73	very occasional stomatocytes
**GLD5**	**II.1**	3.5	M	Hom	p.E1630X	95	N/A	325	N/A	N/A	N/A	N/A	N/A
**GLD6**	**I.1**	27	M	Het	p.R2456C	157	150	350	4.4	5.1	274	1.38	occasional stomatocytes
**GLD6**	**I.2**	21	F	Het	p.F2458L/ p.L939M	129	205	342	3.7	3.9	217	0.73	occasional stomatocytes

Text in red highlights findings outside the normal range. F: female; M: male; N/A, not available; Hom, Homozygous for the mutation; Het, Heterozygous; c-Het, compound heterozygous. Normal range for blood measurements are given in brackets; if two ranges are given, the top is for females, and the bottom for male.

Supplementary Table 2: Summary statistics for exome sequencing - mapping and coverage

	GLD3:II.1	GLD2:II.2	GLD1:II.2
total sequence reads	185330645	90111469	160796040
reads mapped to target	156606033	69988607	122291170
percentage of reads mapped to targets	84.5	77.67	76.05
percentage of reads mapped to targets +/- 150bp	91.69	91.09	83.66
mean coverage	278.46	125.37	217.65
percentage of GENCODE exome covered >=1x	99.43	97.83	97.44
percentage of GENCODE exome covered >=5x	98.26	96.69	94.43
percentage of GENCODE exome covered >=10x	97.05	95.36	92.86
percentage of GENCODE exome covered >=20x	94.92	91.74	90.31

Total number of mapped reads and resulting coverage of the GENCODE exome.

Supplementary Table 3. Summary statistics for exome sequencing – variant calling

	GLD3:II.1		GLD2:II.2		GLD1:II.2	
**Variant type**	**all**	**novel**	**all**	**novel**	**all**	**novel**
All variants	24966	157	24787	198	23844	336
Heterozygous	15167	150	15180	192	13501	306
Homozygous	9799	7	9607	6	10343	30
Coding variants	22063	122	21873	165	21083	292
Heterozygous	13407	121	13461	162	11925	267
Homozygous	8656	1	8412	3	9158	25
Splice variants	2903	35	2914	33	2761	44
Heterozygous	1760	29	1719	30	1576	39
Homozygous	1143	6	1195	3	1185	5
Nonsynonymous SNVs	9939	68	9910	89	9640	164
Heterozygous	6056	68	6130	89	5489	151
Homozygous	3883	0	3780	0	4151	13
Synonymous SNVs	11112	42	11028	53	10633	107
Heterozygous	6766	41	6795	52	5978	98
Homozygous	4346	1	4233	1	4655	9
Stoploss SNVs	14	0	14	0	10	0
Heterozygous	10	0	11	0	4	0
Homozygous	4	0	3	0	6	0
Stopgain SNVs	80	2	80	6	96	5
Heterozygous	58	2	63	6	76	3
Homozygous	22	0	17	0	20	2
Deletions	224	7	225	10	170	9
Heterozygous	134	7	141	10	107	9
Homozygous	90	0	84	0	63	0
Insertions	181	2	183	4	140	3
Heterozygous	97	2	86	2	64	3
Homozygous	84	0	97	2	76	0
Frameshift deletions	68	2	75	6	65	5
Heterozygous	31	2	41	6	37	5
Homozygous	37	0	34	0	28	0
Frameshift insertions	52	0	56	0	47	1
Heterozygous	20	0	23	0	21	1
Homozygous	32	0	33	0	26	0
Transition:Transversion ratio	2.97	2.2	2.88	2	2.92	2.23

Numbers of variants of different classes identified by exome sequencing in the three sequenced cases. SNV - single nucleotide variant.

Supplementary Table 4. Primers for *PIEZO1* (NM_001142864).

Primer Name	Covered exon	Primer Sequence 5'-3'	Primer Name	Covered exon	Primer Sequence 5'-3'
PIEZO1_1F	1	GAGGAAGAGGAGAAGGCGGC	PIEZO1_24F	24	CCACTCACCCAGGCAGAC
PIEZO1_1R	1	CCGCCTTCTCCCAGATTGTCA	PIEZO1_24R	24	CAGCCAGAACAGGTATCGGA
PIEZO1_2F	2	CTCAGGGCAGCACTCACAG	PIEZO1_25F	25	CGTGCCCAACTTTATCCACT
PIEZO1_2R	2	GGGCTGTTAAAGGTGAGGAAA	PIEZO1_25R	25	GGCCACTCACGGTCATAGTA
PIEZO1_3F	3	GATGGGGTCCTGAGTCCATG	PIEZO1_26_27F	26 & 27	CATCATCTCCAAGAACATGCTG
PIEZO1_3R	3	AATCAGGCTGAGTCAACCCC	PIEZO1_26_27R	26 & 27	GCCTGAGACAGTGAGGAAC
PIEZO1_4F	4	GGAGAGGGCCCTGCAGTGA	PIEZO1_28_29F	28 & 29	CAGAGAGGAGCAGGCTGAG
PIEZO1_4R	4	GGTCCCCCTCTGTCCCTCCT	PIEZO1_28_29R	28 & 29	GACGTCACATGGGCACAGA
PIEZO1_5F	5	CCTGGAGACTCATCCACACT	PIEZO1_30F	30	CAAGACGGGAGCACTCAC
PIEZO1_5R	5	AAACAGGTCTGAACGGATGG	PIEZO1_30R	30	AAGGGAGGGGAAGATAGCAG
PIEZO1_6F	6	GAGCAGCTGTGGATGTGGC	PIEZO1_31F	31	CTGCCCACCTCTGTCTTGTC
PIEZO1_6R	6	GTGTCCCGCTGTCTCCACA	PIEZO1_31R	31	GGGCATCTGACACCCTCTAT
PIEZO1_7F	7	CTGGCCGTAACACTGCTTG	PIEZO1_32_33F	32 & 33	CTGAATGGGTGGGAGTCTTC
PIEZO1_7R	7	AAGACCCGTCACAGCCTAC	PIEZO1_32_33R	32 & 33	CACCCTCTGCACCACATG
PIEZO1_8F	8	GGCCATCTCATCTGCCTCTA	PIEZO1_34_35F	34 & 35	CTGGGGAGTGGGAGGTCT
PIEZO1_8R	8	GTCTTGAGCCCAAACCAGC	PIEZO1_34_35R	34 & 35	ACAAGGGAATGGCGAGGG
PIEZO1_9-10F	9 & 10	GTCCTCCTGCTGCTGTGCTA	PIEZO1_36F	36	CACCCTGACCCTCCCTGTAG
PIEZO1_9-10R	9 & 10	CACCGACCCGTGGATGTC	PIEZO1_36R	36	ACACACTGGTACACGGCC
PIEZO1_11F	11	CGGTGTCAGCCCAATTGC	PIEZO1_37F	37	GCTGGTGCCTCTCTGTACC
PIEZO1_11R	11	CTGTGGTAGGTGATGCTCCA	PIEZO1_37R	37	CGACCACCGCGATCTGTG
PIEZO1_12F	12	CTCATCATGGACCAGAGCTATGT	PIEZO1_38F	38	GGCCGTGTACCAGTGTGTG
PIEZO1_12R	12	GGGCAAGTGGACATTGAACA	PIEZO1_38R	38	TAGCACTGAGGGGCGGGA
PIEZO1_13-14F	13 & 14	CTGCTATAGGAGGGTGCTGG	PIEZO1_39F	39	TGGTGAGTGTGAGCCTTGG
PIEZO1_13-14R	13& 14	GCTGTAGTAGACCTGCCGG	PIEZO1_39R	39	GCTTGGCCGTCTCATCTGA
PIEZO1_15F	15	TCTGCCTCACCCTCTTCCA	PIEZO1_40_41F	40 & 41	CAGGGAGAATGCCAGCCT
PIEZO1_15R	15	GAAGGGCCTGTGGAAGTAGT	PIEZO1_40_41R	40 & 41	TGTGGCCAGGAGAGCACAG
PIEZO1_16F	16	ACCGACGAGCAGTGAGTC	PIEZO1_42F	42	CATGTTCCTGGCTGATGTTG
PIEZO1_16R	16	AAGATGTGGCTCCTGTCCAA	PIEZO1_42R	42	GGATGGCCAGGTCAAGGA
PIEZO1_17F	17	AGTTGGACAGGAGCCACATCTT	PIEZO1_43_44F	43 & 44	CCGCCGTCACTGAGAGGT
PIEZO1_17R	17	TGAGGAAGAGGCTCTGGAG	PIEZO1_43_44R	43 & 44	GTTCTCGTGGCAGCAGTG
PIEZO1_18F	18	CTGTGCCATGTCCCAGCTC	PIEZO1_45_46F	45 & 46	TGGGGCTTTTCTGGGCTG
PIEZO1_18R	18	GAAGCGTGGGTAGGGCAG	PIEZO1_45_46R	45 & 46	CAGCTGGGTACAAGTGACAC
PIEZO1_19F	19	GGTGAGTGTGGCAGGCAAC	PIEZO1_47F	47	CCGTCCATCATCCCCTTCAC
PIEZO1_19R	19	CTGGCCCCACTGCACTGAG	PIEZO1_47R	47	GCCCTTCACTCACACAGACTG
PIEZO1_20F	20	TGCCCGTGGTCTCCCTGT	PIEZO1_48F	48	GGAGCTCTACAACGGCAC
PIEZO1_20R	20	CAGGTGGTTCTGCGGAGG	PIEZO1_48R	48	CTGGGATGGAGGGAGAAGATC
PIEZO1_21F	21	TGGGCTACATCCAGGTGAGT	PIEZO1_49_50F	49 & 50	AGTCTGTGTGAGTGAAGGGC
PIEZO1_21R	21	ACCGACACACCACAGGAGTC	PIEZO1_49_50R	49 & 50	AACTTGCCGATGACCAGCAC
PIEZO1_22F	22	ACTCCTGCCCTCTTGACAG	PIEZO1_51F	51	CTGGCTACGGGTGAGTGAG
PIEZO1_22R	22	AGGAGGGTGGACAGGAGT	PIEZO1_51R	51	TGACAGCAGCATCAGGGC
PIEZO1_23F	23	CCTCCTGCTCAGTTCCTTTG			
PIEZO1_23R	23	ACACTTGTGAGCAGATTTGGG			

Supplementary References

1. Andolfo, I. *et al.* Multiple clinical forms of dehydrated hereditary stomatocytosis arise from mutations in PIEZO1. *Blood* **121**, 3925-3935 (2013).
2. Albuisson, J. *et al.* Dehydrated hereditary stomatocytosis linked to gain-of-function mutations in mechanically activated PIEZO1 ion channels. *Nature Communications* **4**(2013).
3. Zarychanski, R. *et al.* Mutations in the mechanotransduction protein PIEZO1 are associated with hereditary xerocytosis. *Blood* **120**, 1908-1915 (2012).
4. Beneteau, C. *et al.* Recurrent mutation in the PIEZO1 gene in two families of hereditary xerocytosis with fetal hydrops. *Clinical Genetics* **85**, 293-295 (2014).
5. Shmukler, B.E. *et al.* Dehydrated stomatocytic anemia due to the heterozygous mutation R2456H in the mechanosensitive cation channel PIEZO1: a case report. *Blood Cells Molecules and Diseases* **52**, 53-54 (2014).
6. Spyropoulos, I.C., Liakopoulos, T.D., Bagos, P.G. & Hamodrakas, S.J. TMRPres2D: high quality visual representation of transmembrane protein models. *Bioinformatics* **20**, 3258-3260 (2004).
